# Health risk assessment of volatile organic compounds (VOCs) in a refinery in the southwest of Iran using SQRA method

**DOI:** 10.3389/fpubh.2022.978354

**Published:** 2022-09-09

**Authors:** Ladan Khajeh Hoseini, Reza Jalilzadeh Yengejeh, Maryam Mohammadi Rouzbehani, Sima Sabzalipour

**Affiliations:** ^1^Department of Environment, Ahvaz Branch, Islamic Azad University, Ahvaz, Iran; ^2^Department of Environmental Engineering, Ahvaz Branch, Islamic Azad University, Ahvaz, Iran

**Keywords:** health risk assessment, volatile organic compounds, refinery, SQRA method, environment

## Abstract

Oil industries, such as oil refineries, are important sources of volatile organic compound production. These compounds have significant health effects on human health. In this study, a health risk assessment is carried out on volatile organic compounds (VOCs) in the recovery oil plant (ROP) unit of a refinery in southwest Iran. It was performed using the SQRA^1^ method including respiratory risk for chronic daily intake (CDI) of VOCs and cancer risk and non-cancer risk indices. Five locations in the area of oil effluents and five locations in the refinery area (control samples) were considered for evaluation. The sampling was done according to the standard NIOSH-1501 and SKC pumps. The gas chromatography/flame ionization detector (GC/FID) method was used to extract VOCs. The cancer slope factor (CSF) and respiratory reference dose (RFC) were calculated in addition to the respiratory risk (CDI). The end result shows that a significant difference was observed between the concentrations of volatile organic compounds in the two groups of air (*P* < 0.05). The SQRA risk assessment showed that the risk levels of benzene for workers in the pit area were very high (4–5). Health hazard levels were also evaluated as high levels for toluene (2–4) and moderate levels for xylene and paraxylene (1–3). The cancer risk assessment of volatile organic compounds recorded the highest level of cancer risk for benzene in the range of petroleum effluents (>1). Also, a non-cancer risk (HQ) assessment revealed that benzene had a significant health risk in the range of oil pits (2–3). Based on the results, petroleum industries, including refineries, should conduct health risk assessment studies of volatile organic compounds. The units that are directly related to the high level of VOCs should be considered sensitive groups, and their employees should be under special management to reduce the level of exposure to these compounds and other hazardous compounds.

## Introduction

Oil and petroleum products are important sources of VOCs. Various research indicates that air pollution in oil centers is significantly more than in other industrial and traffic areas (1). The adverse effects of VOCs on human health have been proven in several studies (2–4). The determination of VOCs in the ambient air of cities and industrial areas is crucial due to the critical effects of volatile organic compounds (VOCs) on living organisms' health and the environment (5, 6). The International Agency for Research on Cancer has classified benzene as a carcinogenic compound for humans (7). VOCs cause hazard risks to human health; assuming how they come into contact with the human body will have different influences on human health (8). Central nervous system reactions such as dizziness, headache, short-term memory loss, eye, nose, and throat irritation, the effects on the respiratory system, the genetic mutations, and the consequent birth of premature infants are the VOC effects reported in various research (9–11). VOCs include a large group of hydrocarbons, which can evaporate at ambient temperature and atmospheric pressure due to their high vapor pressure (12). These components are even able to reduce the ozone formation potential (13). Due to the volatile nature of these compounds, many employees in industrial and nonindustrial environments are exposed to them (14). Respiratory exposure is the most important human contact with this group of compounds (15). Oil refineries are industrial sources that emit VOCs (16). The cancer risk and non-cancer risk of these compounds do not only include employees but also the residents of the refinery area (17). In oil refineries, there is a unit called ROP^2^ operating for the recovery of oil effluent (18). VOCs' evaporation of oil effluents can have significant effects on employees' health and people exposed to the so-called evaporation (19). The adverse effects of chronic exposure to VOCs are divided into two categories: carcinogenic and non-carcinogenic (20). The International Agency for Research on Cancer has classified benzene as a definitive carcinogen (21). Some other VOCs are known as suspected carcinogens, including trichloroethylene, epichlorohydrin, tetrachloroethylene, ethylbenzene, and styrene (22). Today, many international organizations, such as WHO, EPA, and FDA, consider risk assessment as a principle for legislation on chemical compounds (23, 24). Many studies have been performed on the health risk assessment of VOCs in industrial environments. Baltrenas et al. (25) examined the atmospheric BTEX concentrations in an area near a Baltic oil refinery. Zhong et al. (26) estimated the emission factor of VOCs in air profiles of an industrial area such as the Pearl River Delta in China. The increase in VOC concentrations was related to the distance and air currents. Heidari et al. (27) investigated different ways to remove BTEX, on a laboratory scale, from water resources in the oil refinery area of Shiraz. Heibati et al. (28) also assessed the occupational exposure risk of employees in oil derivatives distribution centers with BTEX compounds. In order to study cancer occurrence, Stenehiem et al. (29) considered 25,000 employees in the oil industry especially exposed to high concentrations of benzene. They estimated the incidence of various types of cancer, including lung cancer, was significantly higher than in other control groups. In the non-cancer risk (HQ) study, benzene and toluene had a significant health risk in the oil pit area. Chaiklieng et al. (30) evaluated the non-cancer health risk of benzene at gas stations. In this study, the concentrations of benzene were also more than other VOCs. Currently, there are about 700 oil refineries in the world (31). Bahmanpour et al. (32) studied the environmental risk of air pollutants in industrial towns around Tehran and found that the amount of air pollutants, especially VOCs and PM_10_, are very different from the standard.

The carcinogenesis risk level of employees in 10 oil derivatives distribution centers was unacceptable. The Abadan oil refinery is the largest oil refinery complex with a refining capacity of 430,000 barrels per day (33). However, this oil is extensively isolated by storage tanks and transmission lines; in some parts, such as the ROP unit, a large volume of oil effluents is stored in open pits close to employees. Accordingly, studying the concentration and exposure risk of VOCs in the ROP unit of a refinery containing a large volume of oil effluents is essential. In this study, the level of health risk due to exposure to these compounds in both cancer and non-cancer groups has been investigated by measuring volatile organic compounds at different points within the ROP unit of the refinery.

## Materials and methods

### Study area and ROP unit process

**Figure 1:**
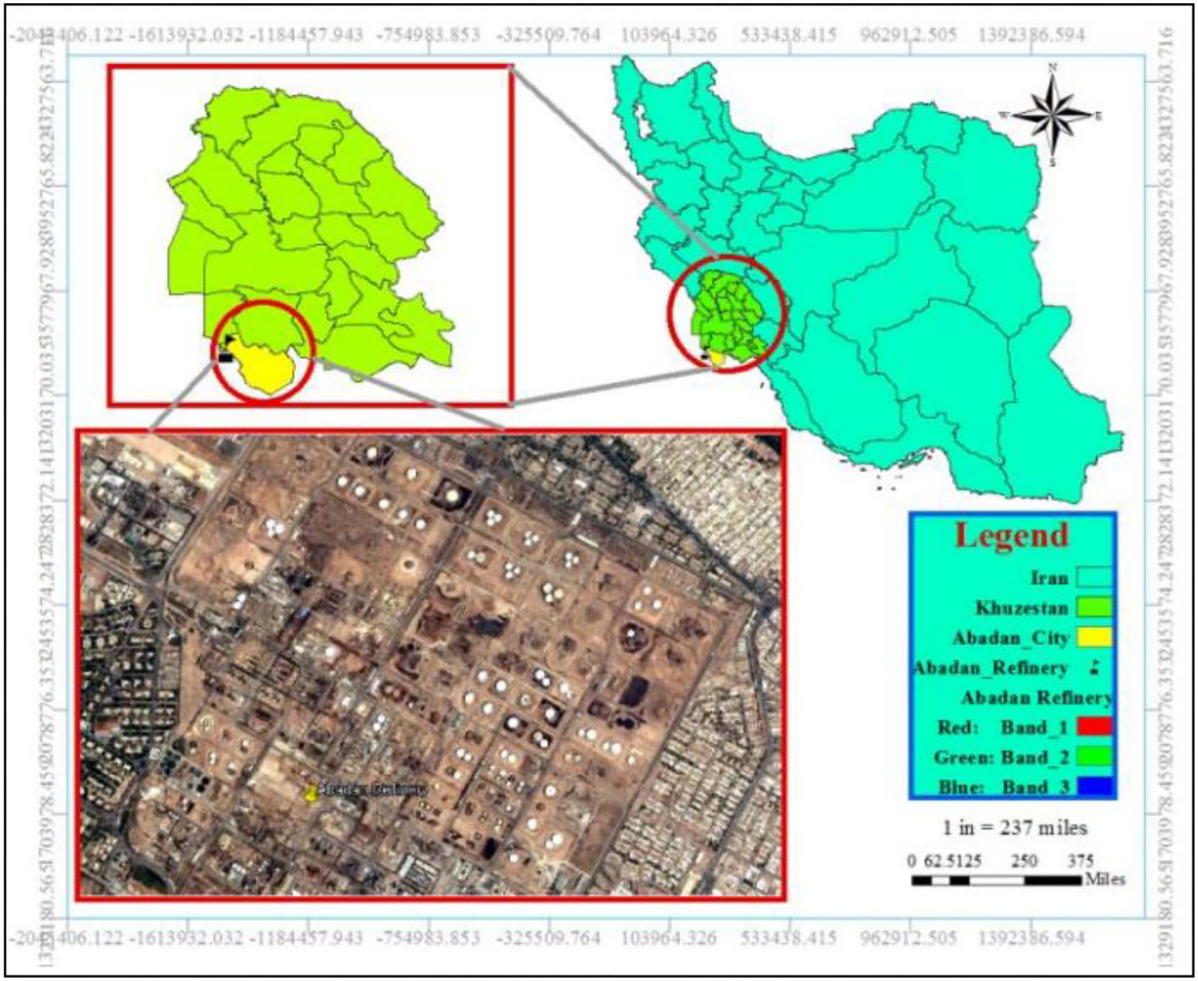
The location of the Abadan oil refinery.

The Abadan oil refinery is located in the center of Abadan, Khuzestan Province, southwest of Iran. The location coordinates are longitude 30.346085 and latitude 48.297848 (Figure 1). It is worthwhile to note that the refinery is close to residential areas.

The effluents of different refinery units are collected through underground cement ditches or pipelines and then within the ROP unit. The unit effluents enter the main separator through 12-, 20-, 24-, 36-, and 54-inch pipes and two cement channels. According to the flow rates that are measured at different times, the flow rate of effluents entering the ROP unit of the Abadan refinery is approximately 350,000 m^3^ per day. At the entry of the ROP unit, the effluents from different units initially enter a pool that makes its content uniform. In this pool, the flow rate, where the flow is laminar, is significantly reduced. On the contrary, during the short retention time considered for the effluent, some suspended solids are settled and become extracted. In addition, mixed oil materials with water have an opportunity to reach the water surface to enter the separation ponds *via* forming oil layers. Due to the high volume of oil effluent in the ROP unit, there are probably high amounts of VOCs. A cross-sectional analysis was performed in the ROP unit of the Abadan oil refinery in January 2020. Taking into consideration the limited area for oil effluent storage pits and the employees exposed to VOCs, five locations in the area of oil effluents and five locations in the refinery area (control samples) were randomly selected for measurement purposes. The number of employees in the ROP unit who were directly exposed to effluent storage pits was 59. The number of samples was calculated based on statistical components (34) and evaluated, considering a type 1 error of 5%. The sampling procedure was performed according to NIOSH-1501 for 1 h using two-part activated carbon pipes and an SKC pump (model 224.44EX) with a flow rate of 0.2 L/min. In this regard, the adsorbent and the sampling pump were placed at the sampling points with a respiratory height of about 1.8 m above the ground. A gas ionization/flame ionization detector (GC/FID) method was used to extract VOCs (35). The VOCs observed in the evaporation of petroleum derivatives were considered. These compounds include benzene, toluene, ethylbenzene, xylene, styrene, n-hexane, p-xylene, and chlorobenzene.

### Exposure risk assessment using the SQRA^3^ method

In the exposure risk assessment using the SQRA method, two factors, including hazard rate (HR) and exposure rate (ER), are important (36). Notably, HR was estimated for each VOC based on previous reports from the World Health Organization and the International Agency for Research on Cancer (21) (Table 1).

**Table 1:** The calculation of the hazard rate for VOCs investigated in this research (19).

**HR rate**	**Description**	**Centers for disease control (CDC)**	**Component**
5	NIOSH recommends as part of its carcinogen policy that the “most protective” respirators be worn for benzene at concentrations above 0.1 ppm.	0.1 ppm	benzene
3	American Conference of Governmental Industrial Hygienists (ACGIH) Threshold Limit Value (TLV): 20 ppm (75 mg/m3)	20 ppm	toluene
2	American Conference of Governmental Industrial Hygienists (ACGIH) Threshold Limit Value (TLV): 100 (435 mg/m3)	100 ppm	ethylbenzene
2	NIOSH Time weighted average (TWA): 100 (435 mg/m3)	100 ppm	xylene
3	NIOSH Time weighted average (TWA): 50 (215 mg/m3)	50 PPM	Styrene
3	NIOSH Time weighted average (TWA): 50 (215 mg/m3)	50 PPM	N-hexane
2	NIOSH Time weighted average (TWA): 100 (435 mg/m3)	100 ppm	P-xlene
4	OSHA Time weighted average (TWA): 10 (46 mg/m3)	10 ppm	Chlorobenzene

The exposure rate also depends on both the VOCs' measured concentrations in the atmospheric area of the ROP unit and the average exposure time for the employees. The exposure rate factor can be calculated using the following equation:


(1) $$\begin{array}{r} E=\frac{F\;\times D\;\times M}{W} \end{array}$$

where *E* is the weekly exposure rate (ppm or mg/m^3^), *F* is the number of employees' attendance at the workplace, *M* is the exposure amount (ppm.mg m^−3^), *D* is the average duration of each exposure (h), and *W* is the average working h of employees per week. It is worthy to note that the value of *E* is calculated and compared with the measured occupational exposure limit (OEL), and the exposure rate is determined in Table 2.

**Table 2:** The exposure ranking calculated based on the exposure rate (34).

**ER = $\frac{\text{E}}{\text{O}E\text{L}}$**	**Exposure ranking**
0.1>	1
0.1–0.5	2
0.51–0.99	3
1–2	4
>2	5

Finally, the risk rate is evaluated based on *HR* and *ER* factors using the following equation (36):


(2) $$\begin{array}{r} \text{Risk level}\;=\;{\left(HR.\;ER\right)}^{\frac{1}{2}} \end{array}$$

The exposure risk of VOCs is evaluated through five levels, including very low, low, medium, high, and very high levels (Table 3).

**Table 3:** Risk prioritization matrix (34).

**Guide**		**probability of occurrence**	**Probability score**	**Impact score**
Little		less than 10%	0.1	0.05
Low		10% to 30%	0.3	0.1
Average		30% to 50%	0.5	0.2
High		50% to 70%	0.7	4
Very high		more than 70%	0.9	0.8

### Inhalation risk calculation

The chronic daily intake *via* inhalation for each investigated VOC is evaluated by the following equation:


(3) $$\begin{array}{r} {\text{CDI}}_{\text{inhal}}=\frac{CA.IR.ET.EF.ED}{BW.AT} \end{array}$$

where CDI_inhal_ is the chronic daily intake of contaminate *via* inhalation (mg kg^−1^day^−1^), *CA* is the concentration of contaminate in air (mg m^−3^), *IR* is the inhalation rate (m^3^ h^−1^), the volume of air inhaled over a specific timeframe, 58.3 mg m^−3^, *ET* is the exposure time (h day^−1^) (the frequency of exposure h per day, 2 h), *EF* is the exposure frequency (days per year), the frequency of exposure (260 working days in a year), and *ED* is the exposure duration (years), which is defined as the time that an individual or population is exposed to contaminants and probably becomes ill. Notably, the average life expectancy, from the time of employment to the end of human life, is estimated at about 50 years. Additionally, BW is body weight (kg) (70 kg on average) and AT includes the number of days a person lives; for the risk assessment of cancer diseases, AT is estimated at about 30 years for urban and industrial environments; it is multiplied by 365 days (equivalent to 8,016 days).

### Cancer risk assessment of VOCs

In cancer risk assessment, even the lowest exposure to the intended contaminant leads to an increased probability of cancer in humans. After the determination of chronic daily intake *via* inhalation (CDI), the cancer risk rate is evaluated by the following equation:


(4) $$\begin{array}{r} \text{Cancer risk}=\text{CDI. CSF} \end{array}$$

CSF is a cancer slope factor calculated for VOCs (Table 4) (37).

**Table 4:** The cancer slope factors for VOCs.

**CSF**	**Component**
5.5 ×10^−2^	Benzene
NA	Toluene
NA	Ethylbenzene
NA	Xylene
1.3 ×10^−2^	Styrene
NA	N-hexane
NA	P-Xylene
NA	Chlorobenzene

### Non-cancer risk assessment

After the determination of the final occupational exposure rate to the hazardous material for ROP employees, the inhalation reference dose (RFDi) factor was used to determine the non-cancer risk rate. Non-cancer risk is displayed by HQ indicia (Eq. 5) (38).


(5) $$\begin{array}{r} {\text{HQ}}_{\text{inhalation}}\;=\;\frac{{CDI}_{inhalation}}{{RfC}_{inhalation<uscore>}} \end{array}$$

If the HQ value is <1 (HQ value <1), contaminates can be acceptable in terms of non-cancer risk. The values of an inhalation reference dose for VOCs are displayed in Table 5.

**Table 5:** The values of RFC for VOCs (35).

**RFC**	**Component**
1 ×10^1^	Benzene
5 ×10^1^	Toluene
1 ×10^3^	Ethylbenzene
2.2 ×10^2^	Xylene
9 ×10^2^	Styrene
7 ×10^2^	N-hexane
2.5 ×10^3^	P-Xylene
5 ×10^1^	Chlorobenzene

## Results

The standard deviations of the measured concentrations of VOCs are shown in Table 6. The results were presented considering the area of the effluent storage pit (five sites) and refinery area (five sites). GC/FID method was applied to determine VOC concentrations reported in μg/m^3^.

**Table 6:** The results of measured VOC concentrations from the ROP unit and Abadan oil refinery area (μg/m^3^).

**Location**	**Benzene**		**Toluene**		**Ethylbenzene**		**Xylene**		**Styrene**		**N-hexane**		**P-xlene**		**Chlorobenzene**	
	**mean**	**S.D**	**mean**	**S.D**	**mean**	**S.D**	**mean**	**S.D**	**mean**	**S.D**	**mean**	**S.D**	**mean**	**S.D**	**mean**	**S.D**
1	8.86	0.21	1.66	0.1	0.26	0.01	1.35	0.12	0.21	0.01	0.01	0	1.48	0.13	0.007	0.001
2	7.53	0.36	1.11	0.06	0.25	0.01	1.3	0.07	0.22	0.02	0.03	0	1.82	0.06	0.004	0
3	9.15	0.25	2.07	0.05	0.32	0.01	1.47	0.09	0.19	0.02	0.02	0	2.19	0.23	0.007	0.001
4	8.03	0.32	1.03	0.05	0.21	0	1.26	0.07	0.25	0.01	0.02	0	1.33	0.09	0.003	0
5	6.9	0.14	0.56	0.02	0.15	0.02	1.1	0.1	0.12	0	0.01	0	1.24	0.05	0.006	0.001
6	2.14	0.07	0.12	0.01	0.04	0	0.34	0.03	0.2	0.02	0.005	0	0.78	0.07	0.00	0
7	3.55	0.15	0.43	0.06	0.04	0	0.58	0.02	0.13	0.01	0.004	0	0.71	0.03	0.00	0
8	1.61	0.09	0.26	0.01	0.02	0	0.31	0.01	0.14	0.01	0.007	0	0.36	0.02	0.001	0
9	2.76	0.11	0.35	0	0.05	0	0.27	0.03	0.18	0.02	0.007	0	0.95	0.06	0.001	0
10	3.1	0.15	0.37	0.04	0.08	0.0‘	0.33	0.02	0.13	0.01	0.01	0	0.28	0.02	0.00	0

**Figure 2:**
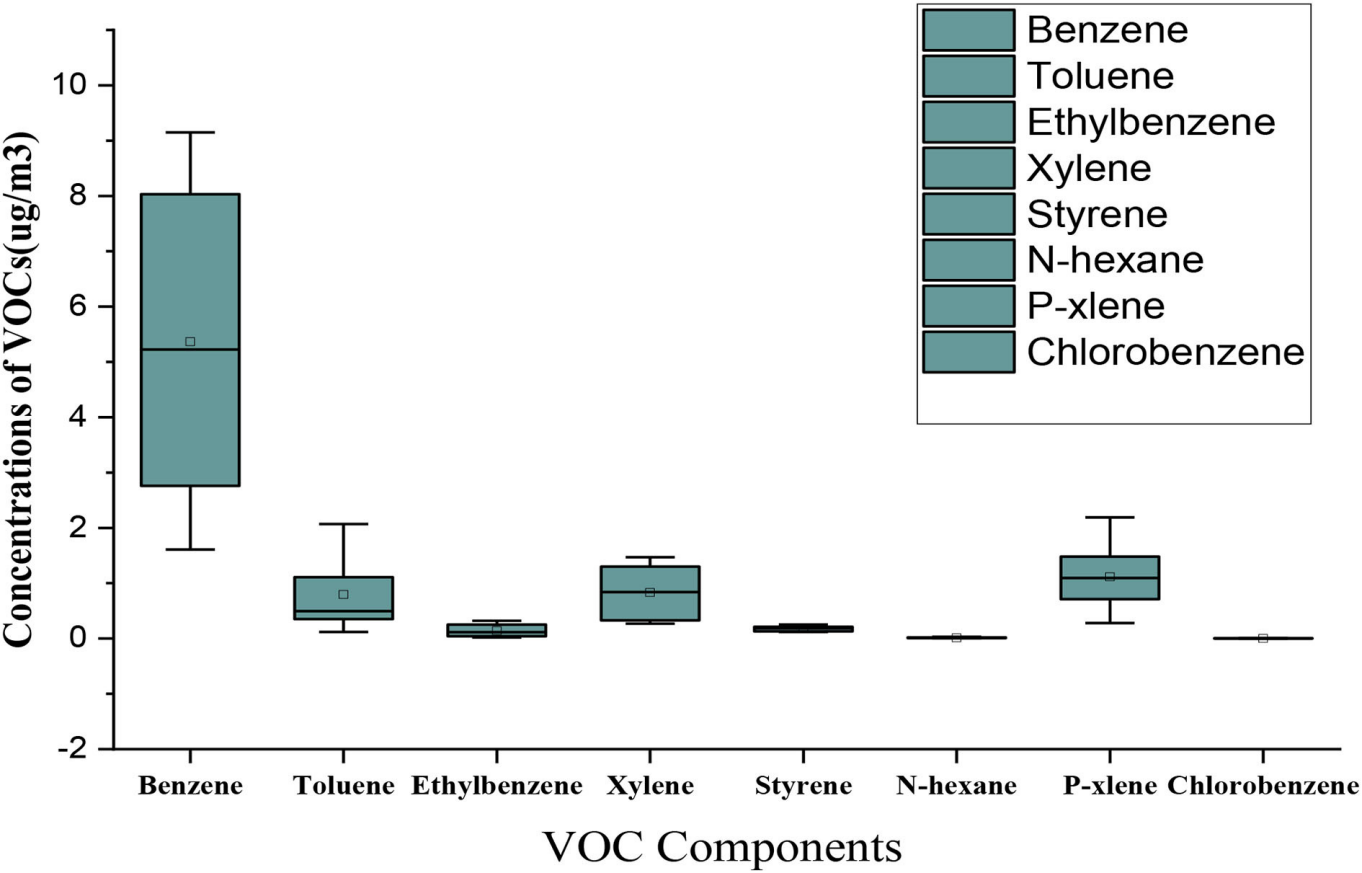
The comparison of the measured concentrations of VOCs in different study areas.

The comparison of the measured concentrations of VOCs based on the type of compounds is displayed in Figure 2. The maximum concentration was related to benzene, toluene, xylene, and p-xylene compounds.

**Figure 3:**
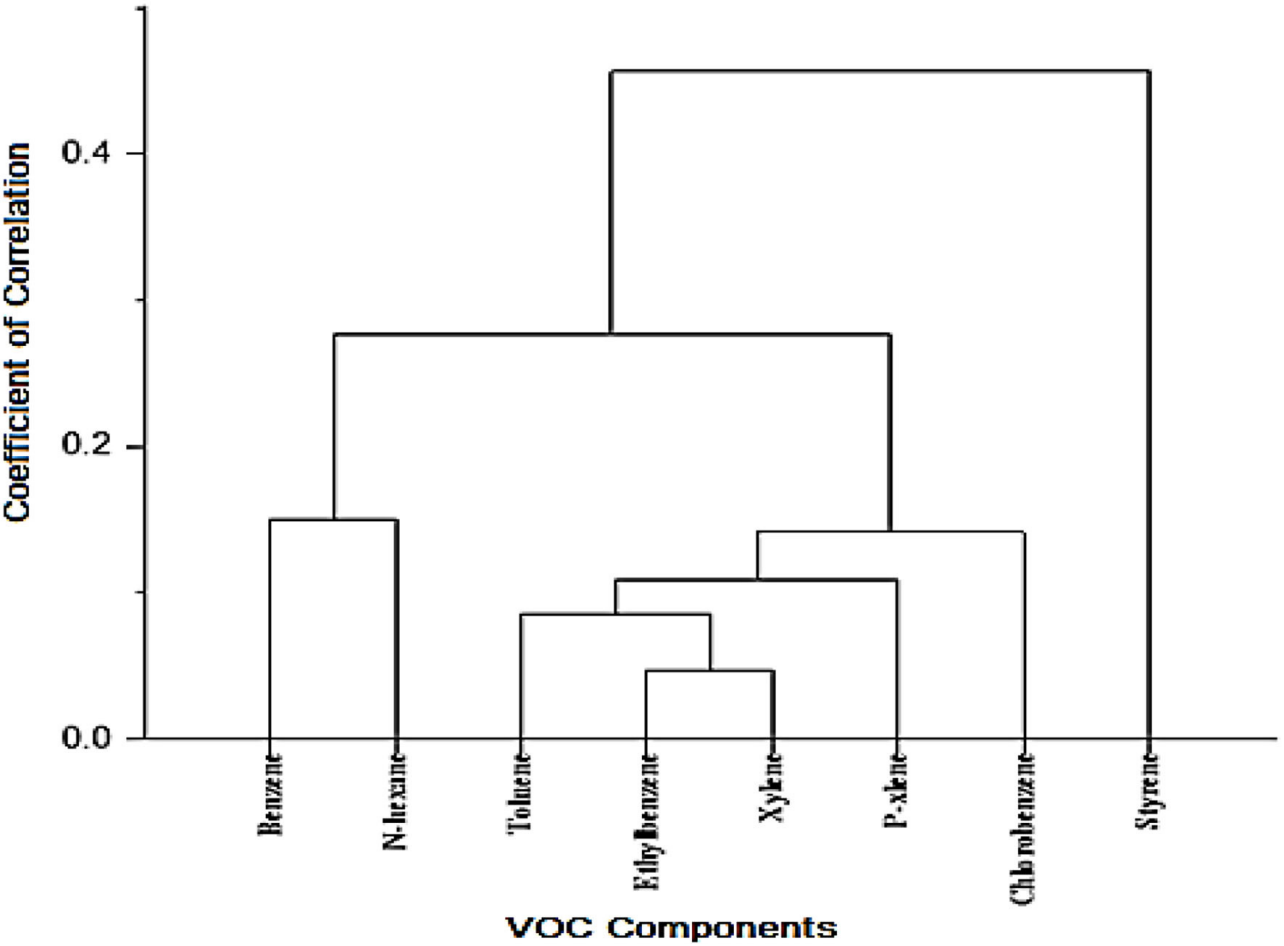
Cluster analysis dendrogram for the correlation between the measured values of volatile organic compounds in the ROP unit of the refinery.

Correlation analysis was used to investigate the origin of volatile organic compounds in the ROP unit (Table 7; Figure 3). The results show that, except for benzene and styrene, there is a significant correlation between the measured values of volatile organic compounds (*p* ≤ 0.05). The concentration of the measured values in different sampling sections has a significant correlation with other compounds that confirms the same origin of these compounds.

**Table 7:** Results of correlation analysis between the concentrations of volatile organic compounds in the ROP unit of the refinery.

		**Benzene**	**Toluene**	**Ethylbenzene**	**Xylene**	**Styrene**	**N-hegane**	**P-xylene**	**Chlorobenzene**
Benzene	Pearson Correlation	1	0.898	0.964	0.986	0.510	0.686	0.881	0.897
	Sig. (two-tailed)		0.000	0.000	0.000	0.132	0.028	0.001	0.000
Toluene	Pearson Correlation		1	0.948	0.881	0.492	0.610	0.866	0.846
	Sig. (two -tailed)			0.000	0.001	0.149	0.061	0.001	0.002
Ethylbenzene	Pearson Correlation			1	0.953	0.561	0.769	0.915	0.874
	Sig. (two -tailed)				0.000	0.092	0.009	0.000	0.001
Xylene	Pearson Correlation				1	0.508	0.719	0.894	0.888
	Sig. (two -tailed)					0.134	0.019	0.000	0.001
Styrene	Pearson Correlation					1	0.592	0.576	0.286
	Sig. (two -tailed)						0.071	0.081	0.422
N_hegane	Pearson Correlation						1	0.733	0.489
	Sig. (two -tailed)							0.016	0.151
P_xylene	Pearson Correlation							1	0.827
	Sig. (two -tailed)								0.003

The comparison of the measured concentrations of VOCs based on studied areas, including the effluent storage pit area (five sites) and refinery area (five sites), was performed using the one-way analysis of variance (*T*-test) method. The results displayed a significant difference between the concentration values of VOCs in the two study areas (*P* <0.05). In fact, VOC concentrations in the atmosphere of the effluent storage pit area were significantly higher than in the ROP area. However, the values of styrene concentration in both studied areas (refinery and pit areas) did not show a statistically significant difference (*P* > 0.05) (Table 8).

**Table 8:** The results of the *T*-test between two sampling areas (pit and refinery areas).

**Sig**.	**Component**
0.000	Benzene
0.006	Toluene
0.000	Ethylbenzene
0.000	Xylene
0.146	Styrene
0.019	N-_hexane
0.002	P-_xlene
0.000	Chlorobenzene

Semi-quantitative risk assessment is a classical method that is used for health risk assessment. The SQRA evaluation is based on the severity of the hazard for each chemical (hazardous level) and the exposure rate. The evaluated values of the health risk assessment of VOCs using the SQRA method are presented in Table 9. The highest risk level (risk level = 5) was related to benzene in sampling site No. 3 in the pit area. The risk level of benzene in all five sampling sites surrounding the pit area was estimated at 4–5 (very hazardous). Additionally, the risk level was estimated at 2–3 (hazardous) for the refinery area samples. The health risk levels evaluated for toluene, xylene, p-xylene, and styrene in the samples of the effluent storage pit area were determined to be 1.4–3.9. The health risk level for n-hexane and chlorobenzene was at least level (risk level =1). The results showed that benzene, toluene, xylene, p-xylene, and ethylbenzene compounds revealed the highest risk level values, while these data showed that n-hexane and chlorobenzene in all sampling areas were non-hazardous. The results also revealed that for the other VOCs, the health risk level in the surrounding pit area was higher than in the refinery area. In addition, it should be noted that, based on the achieved data, health risk management is an essential requisite for the employees working in the effluent pit areas. According to NIOSH, the recommended limit for benzene in long-term exposure is 0.1 ppm, whereas for an 8 h exposure it is 5 ppm (0.016 μg/m^3^). The measured values of benzene concentration in all sampling sites were more than the recommended limits. The recommended limit for toluene in long-term exposure (20 h) and an 8 h exposure is 100 ppm (0.375 μg/m3). Except for sites six and eight (in the ROP area), the concentrations were higher than the recommended limits in other areas. The results showed that the health risk level was high, and at sites 1, 2, and 4, the health risk level was not in an acceptable situation due to the occupational exposure level in the SQRA method at site three (surrounding the effluent storage pit area).

**Table 9:** The health risk assessment of VOCs using SQRA in study sites.

**Location**	**Benzene**	**Toluene**	**Ethylbenzene**	**Xylene**	**Styrene**	**N-hexane**	**P-xlene**	**Chlorobenzene**
1	4.5	3	1.4	1.7	1.4	1	2	1
2	4.5	2	1.4	1.7	1.4	1	2.2	1
3	5	3.9	1.7	1.7	1	1	2.8	1
4	4	2	1	1.7	1.7	1	1.7	1
5	4	1	1	1	1	1	1.4	1
6	2.8	1	1	1	1.4	1	1	1
7	2.8	1	1	1	1	1	1	1
8	2	1	1	1	1	1	1	1
9	3	1	1	1	1	1	1	1
10	2.4	1	1	1	1	1	1	1

The results of the respiratory risk evaluation (CDI, chronic daily intake *via* inhalation) of VOCs of the Abadan refinery ROP unit are summarized in Table 10. The respiratory risk assessment is only based on the amount of compounds inhaled by employees per unit of time, which is not related to the severity of the health hazards of VOCs.

**Table 10:** The respiratory risk evaluations (CDI) of VOCs in study sites.

**Location**	**Benzene**	**Toluene**	**Ethylbenzene**	**Xylene**	**Styrene**	**N-hexane**	**P-xlene**	**Chlorobenzene**
1	23.93	4.484	0.702	3.647	0.567	0.027	3.998	0.019
2	20.34	2.999	0.675	3.512	0.594	0.081	4.917	0.011
3	24.72	5.592	0.864	3.971	0.513	0.054	5.916	0.019
4	21.69	2.782	0.567	3.404	0.675	0.054	3.593	0.008
5	18.64	1.513	0.405	2.972	0.324	0.027	3.35	0.016
6	5.781	0.324	0.108	0.918	0.54	0.014	2.107	0
7	9.59	1.162	0.108	1.567	0.351	0.011	1.918	0
8	4.349	0.702	0.054	0.837	0.378	0.019	0.972	0.003
9	7.456	0.945	0.135	0.729	0.486	0.019	2.566	0.003
10	8.374	1	0.216	0.891	0.351	0.027	0.756	0

**Figure 4:**
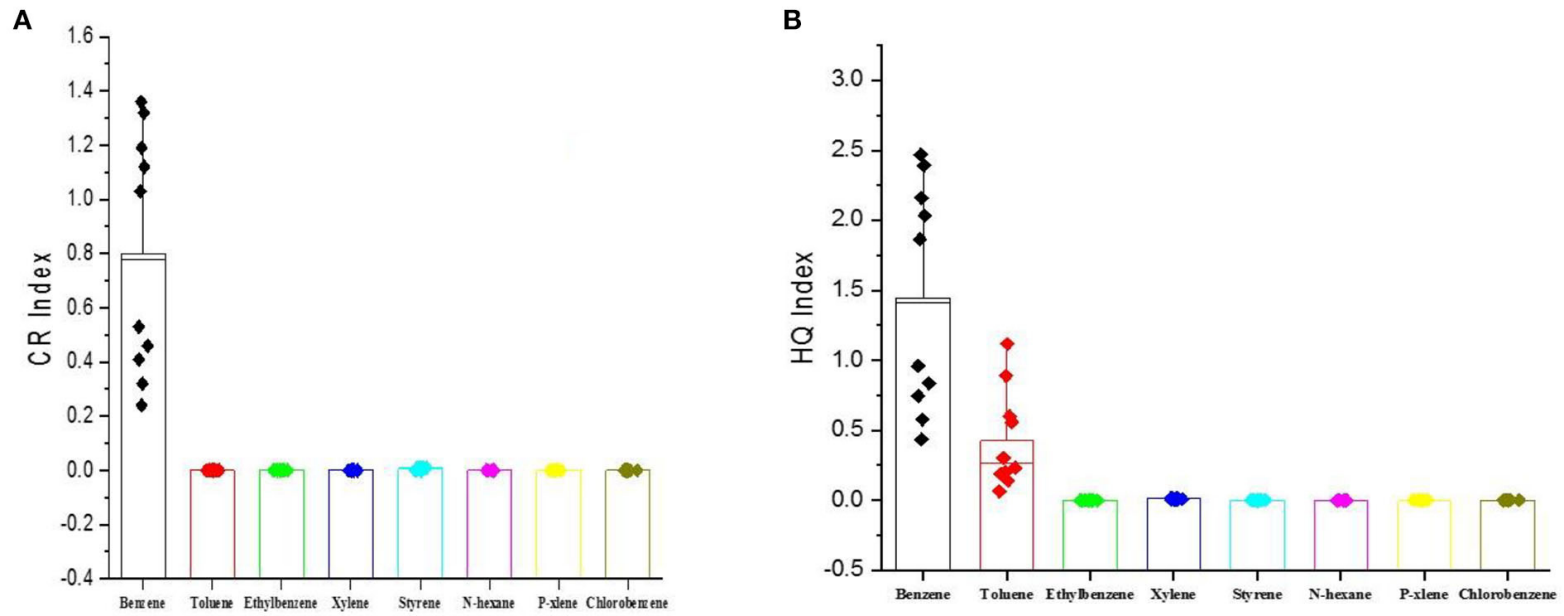
**(A)** The evaluated results for cancer risk (CR) levels. **(B)** The evaluated results for non-cancer.

In order to evaluate cancer and non-cancer levels, a respiratory risk factor (CDI) is required. Cancer and non-cancer risk levels were investigated using cancer slop factor (CSF) and inhalation reference dose (RFCi), respectively. According to research sources' reports such as EPA, NIOSH, and ACGIH, all investigated VOCs in this research have no cancer risk except benzene and styrene. The highest cancer risk levels of benzene were recorded at sites 1–5, which were located around the effluent storage pits (>1). The non-cancer risk level (HQ) for benzene at sites 1–4 was more than two, which, according to the set criteria, is not an acceptable risk level. At site three, the non-cancer risk level for toluene was more than one, which was also a non-acceptable risk level (Table 11, Figure 4). Due to an average uncertainty of about 10% in VOC determination in non-cancer risk assessment, borderline cases (between 0.85 and 1) can also be considered under health risk management.

**Table 11:** The evaluated results for cancer risk (CR) and non-cancer risk (HQ) levels.

	**Benzene**		**Toluene**		**Ethylbenzene**		**Xylene**		**Styrene**		**N-hexane**		**P-xlene**		**Chlorobenzene**	
	**CR**	**HQ**	**CR**	**HQ**	**CR**	**HQ**	**CR**	**HQ**	**CR**	**HQ**	**CR**	**HQ**	**CR**	**HQ**	**CR**	**HQ**
1	1.32	2.393	0	0.89	0	7E-04	0	0.017	0.01	6E-04	0	4E-05	0	0.002	0	4E-04
2	1.12	2.034	0	0.6	0	7E-04	0	0.016	0.01	7E-04	0	1E-04	0	0.002	0	2E-04
3	1.36	2.47	0	1.118	0	9E-04	0	0.018	0.01	6E-04	0	8E-05	0	0.002	0	4E-04
4	1.19	2.16	0	0.556	0	6E-04	0	0.015	0.01	8E-04	0	8E-05	0	0.001	0	2E-04
5	1.03	1.864	0	0.303	0	4E-04	0	0.014	0	4E-04	0	4E-05	0	0.001	0	3E-04
6	0.32	0.578	0	0.065	0	1E-04	0	0.004	0.01	6E-04	0	2E-05	0	8E-04	0	0
7	0.53	0.959	0	0.232	0	1E-04	0	0.007	0	4E-04	0	2E-05	0	8E-04	0	0
8	0.24	0.435	0	0.14	0	5E-05	0	0.004	0	4E-04	0	3E-05	0	4E-04	0	5E-05
9	0.41	0.746	0	0.189	0	1E-04	0	0.003	0.01	5E-04	0	3E-05	0	0.001	0	5E-05
10	0.46	0.837	0	0.2	0	2E-04	0	0.004	0	4E-04	0	4E-05	0	3E-04	0	0

## Discussion

The results of this study showed that the concentration of some volatile organic compounds such as benzene and toluene in the area of the Abadan refinery was significantly higher than the recommended limits reported by international organizations such as NIOSH and ACGIH. While the recommended limit for benzene in long-term exposure is 0.1 ppm, the measured concentrations at some sites in the oil pit area were reported at more than 9 μg/m^3^. The maximum concentrations of toluene, ethyl benzene, and P-Xylene were measured at 2.07, 0.32, and 2.19 μg/m^3^, respectively. These results show that the concentration of these compounds is high in oil refineries. VOC concentrations in the effluent storage pit area in the Abadan refinery ROP unit were significantly higher than their values in the outdoor area.

In the study by Feng et al. (39), benzene and other aromatics and halogenates such as 1,2-dibromoethane, chloromethane, benzene, trichloromethane, and 1,2-dichloroethane contributed approximately 80% of the total carcinogenic risk assessment values in a refinery (39). Afshar et al. (40) reported that the concentration levels of VOCs such as benzene and styrene in the area of oil leakage are significantly high (more than 10 μg/m^3^), which is in accordance with this study data (8–10 μg/m^3^ in oil pit area). Bari et al. (41) emphasized the need for further measurements in oil centers due to the strong relationship between oil derivatives and VOCs. While research has been conducted in the coal mine, the average concentrations of benzene, toluene, and ethyl benzene measured were 3.42, 0.57, and 0.03 μg/m^3^, respectively (42). Notably, the average of these three compounds in the Abadan oil refinery is 5.36, 0.796, and 0.142 μg/m^3^. In a study conducted on the average concentration of benzene in urban areas, its value was reported to be 0.16–0.3 μg/m^3^, which shows a significant difference from the values measured in this study (30). In the paint industry, the average concentrations of the three compounds (benzene, toluene, and ethylbenzene) were performed by researchers, and the average measurements were estimated at 4.32, 1.28, and 0.27 mg/m^3^ (43). The results of a study also showed that exposure to volatile organic compounds such as benzene, ethylbenzene, toluene, and xylene in some businesses, such as nail salons, is beyond the USEPA recommended limit and has a carcinogenic risk (44). Results of this study showed that the average of benzene in the ranking industry is lower than in the refinery, while the amount of toluene is higher than its concentration in the refining industry. It is worth mentioning in this study that various methods were used to determine the health hazard levels of VOCs in the ROP unit of the Abadan oil refinery. In addition, the SQRA method was used as a linear and classical technique to assess the hazard levels of benzene based on the measured concentrations, exposure rate, and the nature of hazards, which were high, especially for employees working in the pit area. The results revealed that health hazard levels for toluene, xylene, and p-xylene were moderate and hazardous. Due to the high concentration of benzene compared to other VOCs, the respiratory risk factor of benzene was estimated to be higher than the other compounds. The results of the cancer risk assessment of VOCs showed that the highest level of cancer risk for benzene was recorded in the effluent storage pit area. Styrene concentration levels were not estimated to be significant in terms of cancer risk. In a petroleum refinery in China, the risk of non-cancer risk was estimated using the US EPA method. The total hazard ratio in the basic chemical area was the highest owing to the highest level of total VOCs. For the cancer risk estimated using the US EPA method, the total cancer risks in all the monitored areas were very high (45). According to the results of this study, the level of non-cancerous risk of benzene in the Abadan oil refinery was calculated to be between 0.435 and 2.47 and cancer risk between 0.24 and 1.36. This showed that the risk levels of benzene for workers in the pit area were unacceptable. Health hazard levels were also evaluated as high for toluene and moderate for xylene and paraxylene. The cancer risk of VOCs recorded the highest level of cancer risk for benzene in the range of petroleum effluents. In a similar study in the paint industry, the carcinogenic risk level of benzene was between 0.27 and 0.38, and its non-cancerous risk was between 0.32 and 0.36, but the non-carcinogenic risk of toluene was estimated to be 3.6–5.1 (46). In another study of large industrial waste transfer stations, the average non-cancerous level of benzene was 0.32 and the risk of cancer was 0.68 (47).

## Data availability statement

The raw data supporting the conclusions of this article will be made available by the authors, without undue reservation.

## Author contributions

LK: conceptualization and performed the study design, literature review, and experiments. RJ: data analysis and interpretation. MM: involved in the study design, review and editing, and project administration. SS: study design and review and editing. All authors contributed to the article and approved the submitted version.

## Conflict of interest

The authors declare that the research was conducted in the absence of any commercial or financial relationships that could be construed as a potential conflict of interest.

## Publisher's note

All claims expressed in this article are solely those of the authors and do not necessarily represent those of their affiliated organizations, or those of the publisher, the editors and the reviewers. Any product that may be evaluated in this article, or claim that may be made by its manufacturer, is not guaranteed or endorsed by the publisher.

## Footnotes

^2^Recovery oil plant.

^3^Semi-quantitative risk assessment.
